# Evaluating GPT-4o for emergency disposition of complex respiratory cases with pulmonology consultation: a diagnostic accuracy study

**DOI:** 10.1186/s13049-025-01475-3

**Published:** 2025-10-02

**Authors:** Cem Yıldırım, Ahmet Aykut, Ertuğ Günsoy, Mehmet Veysel Öncül

**Affiliations:** Department of Emergency Medicine, Van Education and Research Hospital, Van, Türkiye

**Keywords:** Emergency department, GPT-4o, Clinical decision support, Large language models, Disposition prediction, Pulmonary conditions, AI in medicine

## Abstract

**Background:**

Large Language Models (LLMs), such as GPT-4o, are increasingly investigated for clinical decision support in emergency medicine. However, their real-world performance in disposition prediction remains insufficiently studied. This study evaluated the diagnostic accuracy of GPT-4o in predicting ED disposition—discharge, ward admission, or ICU admission—in complex emergency respiratory cases requiring pulmonology consultation and chest CT, representing a selective high-acuity subgroup of ED patients.

**Methods:**

We conducted a retrospective observational study in a tertiary ED between November 2024 and February 2025. We retrospectively included ED patients with complex respiratory presentations who underwent pulmonology consultation and chest CT, representing a selective high-acuity subgroup rather than the general ED respiratory population. GPT-4o was prompted to predict the most appropriate ED disposition using three progressively enriched input models: Model 1 (age, sex, oxygen saturation, home oxygen therapy, and venous blood gas parameters); Model 2 (Model 1 plus laboratory data); and Model 3 (Model 2 plus chest CT findings). Model performance was assessed using accuracy, sensitivity, specificity, positive predictive value (PPV), negative predictive value (NPV), and F1 score.

**Results:**

Among the 221 patients included, 69.2% were admitted to the ward, 9.0% to the intensive care unit (ICU), and 21.7% were discharged. For hospital admission prediction, Model 3 demonstrated the highest sensitivity (91.9%) and overall accuracy (76.5%), but the lowest specificity (20.8%). In contrast, for discharge prediction, Model 3 achieved the highest specificity (91.9%) but the lowest sensitivity (20.8%). Numerical improvements were observed across models, but none reached statistical significance (all *p* > 0.22). Model 1 therefore performed comparably to Models 2–3 while being less complex. Among patients who were discharged despite GPT-4o predicting admission, the 14-day ED re-presentation rates were 23.8% (5/21) for Model 1, 30.0% (9/30) for Model 2, and 28.9% (11/38) for Model 3.

**Conclusion:**

GPT-4o demonstrated high sensitivity in identifying ED patients requiring hospital admission, particularly those needing intensive care, when provided with progressively enriched clinical input. However, its low sensitivity for discharge prediction resulted in frequent overtriage, limiting its utility for autonomous decision-making. This proof-of-concept study demonstrates GPT-4o’s capacity to stratify disposition decisions in complex respiratory cases under varying levels of limited input data. However, these findings should be interpreted in light of key limitations, including the selective high-acuity cohort and the absence of vital signs, and require prospective validation before clinical implementation.

**Supplementary Information:**

The online version contains supplementary material available at 10.1186/s13049-025-01475-3.

## Introduction

Large Language Models (LLMs), such as GPT-4o, have emerged as promising tools for supporting clinical decision-making in emergency medicine [1]. By processing complex, unstructured clinical data, these models can assist with diagnostic reasoning, triage classification, and patient disposition—key elements of emergency department (ED) workflows.

Early studies have shown that LLMs, including GPT-4, can outperform resident physicians in diagnostic accuracy across simulated ED scenarios [2]. In triage support, these models have demonstrated moderate agreement with experienced triage staff, achieving approximately 76% accuracy and over 95% specificity for high-acuity classifications [3]. GPT-4 has also exhibited substantial inter-rater reliability (Cohen’s κ ≈ 0.65–0.67) in acuity level assignments, comparable to junior clinicians without formal triage training [4].

Despite these promising results, several limitations have emerged. A recent systematic review reported wide variability in ChatGPT’s triage performance, with some studies showing level assignment accuracy as low as 50% [5]. Additionally, large-scale evaluations of GPT-4 suggest a consistent conservative bias, characterized by high sensitivity but low specificity in disposition predictions—frequently overestimating the need for admission compared to emergency physicians [1].

GPT-4o, the latest generation of OpenAI’s LLMs, introduces improved processing capabilities and lower latency, potentially increasing its clinical applicability. However, its real-world performance in complex, specialty-referred ED cases—particularly for disposition decisions—remains largely untested.

This study aimed to evaluate the diagnostic accuracy of GPT-4o in predicting ED disposition outcomes—discharge, ward admission, or ICU admission—among adult patients with isolated pulmonary conditions who were evaluated by a pulmonologist. Using real patient data, we tested three structured input models of increasing clinical richness to assess whether GPT-4o could replicate actual clinical decisions in a stratified decision-support framework.

## Methods

### Study design and setting

This retrospective observational study was conducted in the ED of a large tertiary academic hospital in Türkiye between November 1, 2024, and February 28, 2025. The aim was to evaluate the diagnostic performance of GPT-4o, a general-purpose LLM developed by OpenAI, in predicting ED disposition outcomes—discharge, ward admission, or ICU admission—using real-world clinical data. The study focused on adult patients with isolated pulmonary conditions who were evaluated by the pulmonology department following an ED consultation.

All clinical data were fully anonymized before analysis. No patient names, identifiers, visit timestamps, or personally identifiable information were included in any of the prompts submitted to GPT-4o. Data were pre-processed locally in a secure environment and manually reviewed to ensure de-identification.

The study protocol was approved by the Clinical Research Ethics Committee of Van Education and Research Hospital (Approval No: GOKAEK/2025-04-09), and the requirement for informed consent was waived due to the retrospective and de-identified nature of the dataset.

### Participants

Inclusion was limited to consecutive ED patients who underwent pulmonology consultation and chest CT. This approach ensured focus on complex respiratory cases requiring specialist input, not unselected ED patients. To reflect real-world clinical workflow, a consecutive sampling strategy was applied. Inclusion criteria required complete clinical data, including oxygen saturation (SpO₂), venous blood gas parameters, relevant laboratory results, and chest CT findings.

In our institution, all chest CT scans were ordered by emergency physicians either prior to or concurrently with pulmonology consultation. Pulmonologists did not independently initiate imaging.

Patients who underwent either conventional chest CT or CT pulmonary angiography were eligible, provided that all other inclusion criteria were met.

Patients were excluded if they had missing key data elements, underwent multiple specialty consultations beyond pulmonology, or were diagnosed with pulmonary embolism (PE), as such cases are automatically admitted at our institution and would introduce bias in disposition prediction. Patients who underwent CT pulmonary angiography and were found not to have embolism were included only if they met all other inclusion criteria and were managed solely by the pulmonology department.

The study population included patients with isolated non-embolic pulmonary conditions, such as pneumonia, chronic obstructive pulmonary disease (COPD) exacerbations, bronchiectasis, and interstitial lung disease. After applying these criteria, a total of 221 unique patient cases were included in the final analysis.

This study focused on patients who required both chest CT imaging and pulmonology consultation, reflecting a subset with higher clinical complexity and diagnostic uncertainty as judged by the treating emergency physicians.

### Data collection and variables

Clinical data were retrospectively extracted from the hospital’s electronic health record system. The collected variables included age, sex, oxygen saturation (SpO₂, %) recorded at initial triage, long-term home oxygen therapy status, venous blood gas parameters (pH, pCO₂ in mmHg, HCO₃ and base excess in mEq/L, lactate in mmol/L), laboratory markers (white blood cell count [WBC] in 10³/µL, C-reactive protein [CRP] in mg/L, urea and creatinine in mg/dL), and chest CT report summaries.

For patients receiving long-term home oxygen therapy, SpO₂ was measured while on supplemental oxygen; for others, values reflected room air readings. Chest CT findings were extracted directly from formal radiology reports without reinterpretation.

All data underwent structured preprocessing and quality checks. Continuous variables were reviewed for physiological plausibility, and extreme outliers were flagged for manual verification. Cases with incomplete blood gas or CT data were excluded.

The final clinical disposition—discharge, ward admission, or ICU admission—was confirmed via the hospital’s admission records and served as a proxy for real-time clinical decision-making. Although timestamped decisions from individual providers were not available, disposition outcomes reflected the pulmonologist’s documented judgment following consultation in the ED.

### Prediction procedure

Each patient case was processed through GPT-4o using structured prompts representing three progressively enriched clinical input levels:

- **Model 1**: Age, sex, SpO₂, long-term home oxygen therapy status, and venous blood gas parameters (pH, pCO₂, HCO₃, lactate, base excess).
- **Model 2**: Model 1 plus laboratory values (WBC, CRP, urea, creatinine).
- **Model 3**: Model 2 plus a summary of chest CT findings from the radiology report.

Prompts were constructed using a fixed standardized template, with patient-specific variables inserted into predefined text slots. All prompts began with the same instruction:

Based on the following patient data, please determine the most appropriate ED disposition: discharge, ward admission, or ICU admission.

Each prompt was submitted manually in an isolated session to ensure stateless interaction and eliminate contextual memory effects. This approach effectively blinded the model to other patient data and preserved prediction independence. No feedback loops, follow-up clarifications, or parameter tuning were applied.

To ensure consistency, all prompts were submitted between May 5 and May 10, 2025, using the same deployed version of GPT-4o available through OpenAI’s public interface. No post-hoc edits or reinterpretations were made to model outputs. Detailed prompt structures are presented in Supplementary Table 1.

### Outcomes and definitions

The primary outcome was the diagnostic accuracy of GPT-4o in predicting each patient’s clinical disposition category—namely discharge, ward admission, or ICU admission—based on hospital admission records. Each model output was matched to the actual disposition label, and classified as correct (true positive or true negative) or incorrect (false positive or false negative) accordingly. Predictions were considered correct if the GPT-4o output exactly matched the real-world disposition.

The secondary outcome was the 14-day ED re-presentation rate among patients who were discharged in real life but predicted as “admit” (ward or ICU) by GPT-4o. This metric served as a proxy to assess whether false-negative discharge decisions by clinicians (and “true positive” GPT predictions) were potentially clinically meaningful.

### Statistical analysis

Descriptive statistics were used to summarize patient demographics and clinical parameters across disposition categories. One-way ANOVA was used for normally distributed continuous variables, the Kruskal–Wallis test for non-normally distributed continuous variables, and chi-square or Fisher’s exact test for categorical variables. In addition to p-values, standardized mean differences (SMDs) were calculated to quantify effect sizes. For each GPT-4o input model (Models 1–3), diagnostic performance was evaluated using standard classification metrics: accuracy, sensitivity, specificity, positive predictive value (PPV), negative predictive value (NPV), and F1 score.

Model predictions were considered correct if they exactly matched the patient’s actual clinical disposition (i.e., discharge, ward admission, or ICU admission). Confusion matrix components were calculated for each disposition category.

To compare predictive performance across the three input models, both descriptive trends and inferential statistics were employed. Specifically:

- McNemar’s exact test was used to evaluate pairwise differences in classification accuracy.
- Bootstrap resampling (*n* = 10,000) was applied to estimate confidence intervals for differences in accuracy.
- Permutation testing (*n* = 10,000) was conducted to assess differences in F1 scores between models.

Given the relatively small number of ICU admissions (*n* = 20), no subgroup or multivariable analyses were performed. All statistical analyses were performed using Jamovi (version 2.6.26) and Python 3.10 (scikit-learn, numpy, matplotlib). Detailed results of all comparative statistical tests are presented in Supplementary Table 2.

### Sample size justification

The expected sensitivity of GPT-4o for hospital admission prediction was estimated at 80%, based on pilot testing and early diagnostic accuracy benchmarks from LLMs in emergency medicine. Power analysis indicated that a minimum of 59 cases would be required to achieve 80% power with a ± 10% precision margin at a 95% confidence level.

However, to ensure robust evaluation across all disposition categories—including the less frequent ICU admissions—and to maintain external validity, a consecutive sampling strategy was used. All eligible patients during the study period were included, resulting in a final analytic sample of 221 cases. This approach allowed for stratified comparisons across the three input models and preserved the real-world distribution of outcomes.

## Results

### Study population

**Fig. 1:**
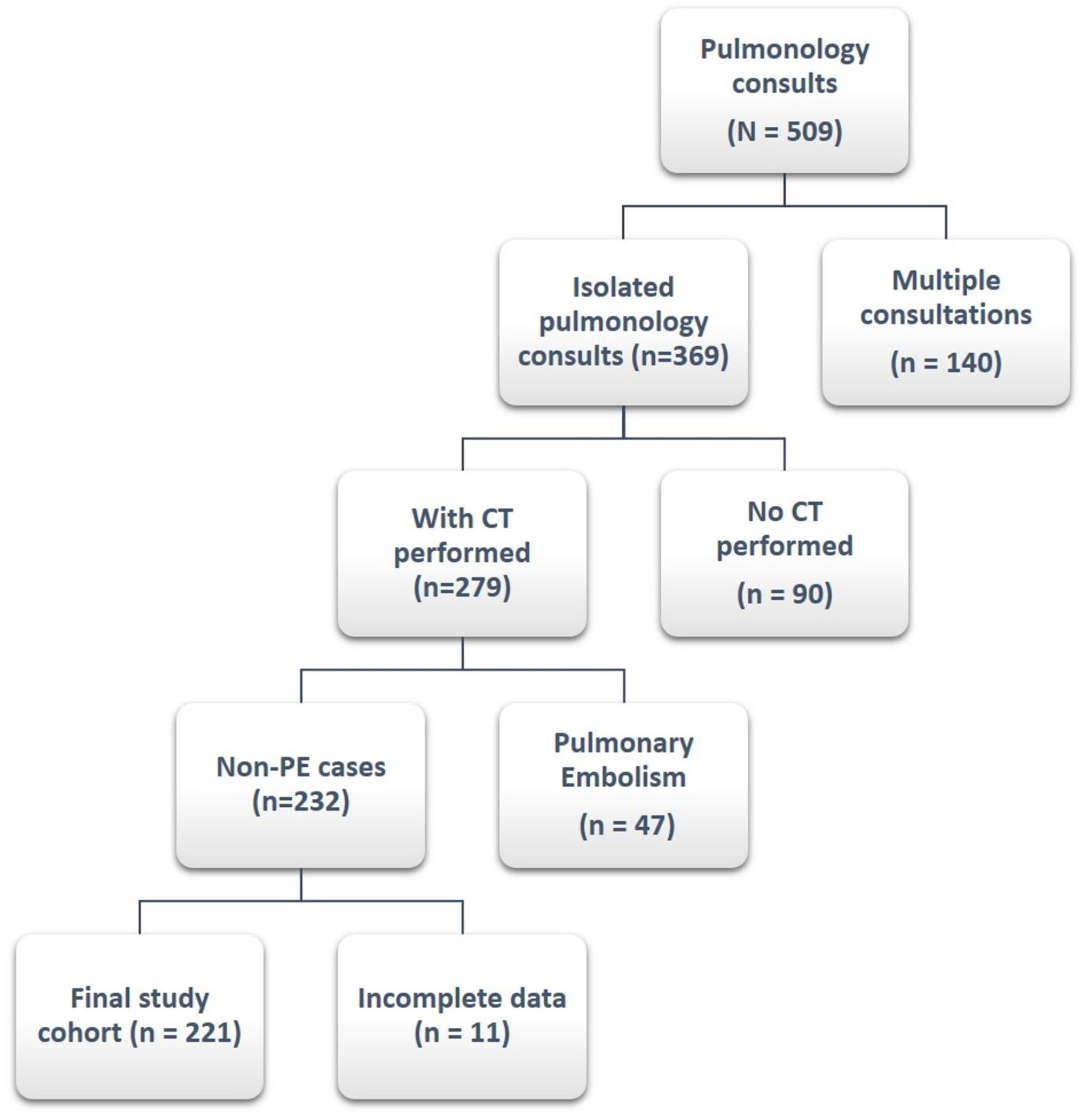
Flow diagram of patient selection. Of 509 pulmonology consultations, 140 patients with multiple consultations were excluded. Among the remaining 369 isolated consultations, 90 without chest CT were excluded. Of 279 patients with CT performed, 47 with pulmonary embolism and 11 with incomplete data were excluded. The final study cohort consisted of 221 non-PE patients

A total of 221 adult patients with isolated pulmonary conditions who were evaluated by the pulmonology department upon consultation from the ED between November 1, 2024, and February 28, 2025, were included in the analysis. The flow of patient selection and exclusions is shown in Fig. 1. The mean age of the study population was 67.9 ± 14.9 years, and 46.6% were male. Regarding clinical disposition, 153 patients (69.2%) were admitted to the general ward, 20 patients (9.0%) to the ICU, and 48 patients (21.7%) were discharged directly from the ED. The diagnostic distribution is shown in Table 1: pneumonia (40.3%), COPD (21.3%), and COPD + pneumonia overlap (30.3%) were most frequent, followed by interstitial lung disease (6.8%) and bronchiectasis (1.4%).

**Table 1:** Distribution of primary diagnoses in the study population (*n* = 221)

Diagnosis Category	*n*	%
Isolated pneumonia	89	40.3
Isolated COPD	47	21.3
COPD + pneumonia	67	30.3
Interstitial lung disease	15	6.8
Bronchiectasis	3	1.4
Total	221	100

Note: A small proportion of patients with interstitial lung disease or bronchiectasis may also have had superimposed pneumonia. For clarity of presentation, these patients were categorized under their primary chronic lung disease

### Clinical and laboratory characteristics by disposition

Table 2 summarizes key demographic, clinical, and laboratory variables by final disposition category. Statistically significant differences were observed in SpO₂, pH, and pCO₂ values across disposition groups, with large effect sizes. The median oxygen saturation was 90.0% in discharged patients, 80.0% in ward admissions, and 70.0% in ICU admissions (*p* < 0.001, SMD up to 2.05 for Discharge vs. ICU). Similarly, median pH values were 7.38, 7.39, and 7.28, respectively (*p* < 0.001, SMD up to 0.87), and median pCO₂ values were 44.0 mmHg, 45.4 mmHg, and 67.8 mmHg, respectively (*p* < 0.001, SMD up to 1.47). No statistically significant differences were found in HCO₃, lactate, base excess, CRP, WBC, urea, or creatinine levels (all *p* > 0.05, all SMD < 0.2).

**Table 2:** Demographic, clinical, and laboratory characteristics of the study population

Characteristic	Total (221)		Discharged (48)		Ward (153)		ICU (20)		*p*-value
**Age (Mean**,** SD)**	**67.9**	(14.9)	**67.1**	16.4	**67.5**	14.5	**73.5**	14.0	0.222
**Gender (M/F)**,** n (%)**	**103/118**	46.6%	**24/24**	50%	**67/86**	43.8%	**12/8**	60%	0.341
**Home Oxygen Therapy**	**79**	35.7%	**14**	29.2%	**54**	35.3%	**11**	55.0%	0.126
**SpO₂ (%) (Median**,** Q1-Q3)**	**80**	70–88	**90**	80–94	**80**	70–86	**70**	60–74	**< 0.001**
**Blood Gas**	**Median**	**Q1-Q3**	**Median**	**Q1-Q3**	**Median**	**Q1-Q3**	**Median**	**Q1-Q3**	
**pH**	**7.38**	7.35–7.43	**7.38**	7.36–7.42	**7.39**	7.36–7.44	**7.28**	7.23–7.33	**< 0.001**
**pCO₂ (**mmHg)	**45.8**	40-53.5	**44.0**	40.4–52.5	**45.4**	39.1–51.8	**67.8**	58.0-76.3	**< 0.001**
**HCO₃ (**mEq/L)	**25.7**	23.6–27.3	**25.4**	23.7–27.2	**25.7**	23.8–27.3	**25.9**	21.0-27.4	0.715
**Lactate (**mmol/L)	**1.90**	1.40–2.60	**1.70**	1.30–2.25	**1.90**	1.50–2.60	**1.85**	1.30–3.42	0.197
**Base Excess (**mEq/L)	**2.90**	0.20–5.30	**2.60**	0.25–5.03	**3.10**	0.20–5.10	**4.30**	0.75–8.13	0.477
**Laboratory**	**Median**	**Q1-Q3**	**Median**	**Q1-Q3**	**Median**	**Q1-Q3**	**Median**	**Q1-Q3**	
**WBC (**10³/µL)	**10.1**	7.43–14.1	**9.13**	7.08–12.1	**10.7**	7.23–14.2	**9.20**	8.49–13.8	0.278
**CRP (**mg/L)	**45.4**	14.0-119	**29.5**	13.7–72.5	**52.3**	16.9–137	**21.0**	9.05–68.8	0.060
**Urea (**mg/dL)	**38.6**	29.4–53.5	**38.6**	31.2–49.2	**37.3**	28.5–53.1	**48.8**	36.7–65.8	0.107
**Creatinine(**mg/dL)	**0.82**	0.67–1.10	**0.83**	0.66–1.08	**0.82**	0.68–1.10	**0.98**	0.66–1.24	0.633

Demographic, clinical, and laboratory characteristics of 221 patients, stratified by final disposition (discharge, ward, ICU). Values are reported as mean ± SD or median (Q1–Q3), depending on distribution

Statistical comparisons across the three disposition groups were performed using one-way ANOVA for normally distributed continuous variables (e.g., age), Kruskal–Wallis test for non-normally distributed continuous variables (e.g., SpO₂, pH, pCO₂, HCO₃, lactate, base excess, CRP, WBC, urea, creatinine), and chi-square or Fisher’s exact test for categorical variables (e.g., sex, home oxygen therapy). A p-value < 0.05 was considered statistically significant

In addition to p-values, standardized mean differences (SMDs) were calculated to quantify effect sizes. Large SMDs were observed for SpO₂, pH, and pCO₂, consistent with clinically meaningful differences between disposition groups, whereas all other variables had negligible effect sizes (SMD < 0.2)

### Model performance for hospital admission and discharge

The diagnostic performance of GPT-4o across three input models is presented in Tables 3 and 4. For overall hospital admission (ward or ICU), model accuracy increased from 72.4% in Model 1 to 76.5% in Model 3. Sensitivity also improved from 76.9 to 91.9%, while specificity declined from 56.2 to 20.8%, indicating a shift toward higher sensitivity at the cost of increased false-positive predictions.

**Table 3:** Diagnostic performance of GPT-4o-based disposition predictions across three levels of clinical input

Category	Model	Accuracy	Sensitivity	Specificity	PPV	NPV	TP	TN	FP	FN
**Admission** **Ward + ICU**	**1**	72.4	76.9	56.2	86.4	40.3	133	27	21	40
	**2**	75.6	86.1	37.5	83.2	42.9	149	18	30	24
	**3**	76.5	91.9	20.8	80.7	41.7	159	10	38	14
**Ward**	**1**	53.4	48.4	64.7	75.5	35.8	74	44	24	79
	**2**	57.9	61.4	50	73.4	36.6	94	34	34	59
	**3**	60.2	69.9	38.2	71.8	36.1	107	26	42	46
**ICU**	**1**	79.2	75	79.6	26.8	97.0	15	160	41	5
	**2**	80.5	70	81.6	27.5	96.5	14	164	37	6
	**3**	81.9	70	83.1	29.2	96.5	14	167	34	6

Diagnostic performance of GPT-4o in predicting hospital, ward, and ICU admissions across three input models. Metrics include accuracy, sensitivity, specificity, PPV, NPV, and confusion matrix values (TP, TN, FP, FN)

**Table 4:** Diagnostic performance of GPT-4o-based models in predicting patient discharge

Model	Accuracy	Sensitivity	Specificity	PPV	NPV	TP	TN	FP	FN
**1**	72.4	56.2	76.9	40.3	86.4	27	133	40	21
**2**	75.6	37.5	86.1	42.9	83.2	18	149	24	30
**3**	76.5	20.8	91.9	41.7	80.7	10	159	14	38

GPT-4o’s performance in predicting discharge across three input models. Includes accuracy, sensitivity, specificity, PPV, NPV, and confusion matrix values (TP, TN, FP, FN)

For discharge prediction, Model 1 demonstrated relatively balanced metrics (sensitivity 56.2%, specificity 76.9%), while Model 3 achieved higher specificity (91.9%) but lower sensitivity (20.8%), suggesting a pattern of overtriage in more complex input models.

### F1 score comparison across models

Table 5 presents the F1 scores across disposition outcomes. Model 3 yielded the highest F1 scores for hospital admission (0.859) and ward admission (0.709), while ICU-related scores remained relatively consistent across all models (approximately 0.39–0.41). Model 1 had the lowest scores for hospital (0.813) and ward admission (0.590), whereas Model 2 provided intermediate performance values. These results indicate modest gains in predictive balance with increasing clinical input complexity, particularly in non-critical disposition categories.

**Table 5:** F1 scores of GPT-4o-based models across clinical disposition outcomes

Model	Admission	Ward	ICU
**1**	0.813	0.590	0.395
**2**	0.847	0.669	0.394
**3**	0.859	0.709	0.412

F1 scores of GPT-4o across models and disposition outcomes (hospital, ward, ICU), representing the balance of sensitivity and precision

Although Model 3 yielded the highest performance across most categories, no statistically significant differences were observed between any model combinations (all *p* > 0.22).

Pairwise comparisons of model accuracy produced confidence intervals that included zero, and permutation testing showed higher median F1 scores for Model 3 that did not reach statistical significance (all *p* > 0.97).

### Subgroup analysis by age and oxygen saturation

In subgroup analyses, accuracy was comparable between younger and older patients (< 65 years: 68.1–76.4%; ≥65 years: 75.2–77.9%). The age cut-off of 65 years was chosen as it represents a conventional clinical threshold frequently used in respiratory and critical care research. By contrast, performance varied by oxygen saturation: in patients with SpO₂ <80%—corresponding to the median oxygenation value of the study cohort—accuracy was high (92.9%) due to maximal sensitivity (100.0%) and minimal specificity (0.0%). In those with SpO₂ ≥80%, accuracy was more modest (56.6–63.9%), with a sensitivity–specificity trade-off across models (sensitivity 51.2%→82.9%, specificity 67.5%→25.0%) (see Supplementary Table 3).

### Emergency department Re-presentations after discharge

Among the 48 patients who were discharged from the ED, GPT-4o recommended admission in 21 cases using Model 1, 30 cases using Model 2, and 38 cases using Model 3. These cases were considered false-positive admissions (model-recommended admission but actual discharge). Fourteen-day return rates were 23.8% (5/21) for Model 1, 30.0% (9/30) for Model 2, and 28.9% (11/38) for Model 3. Diagnoses among returners were predominantly pneumonia (Model 1: 40%; Model 2: 66.7%; Model 3: 72.7%), followed by COPD, COPD + pneumonia, and interstitial lung disease (Supplementary Table 4).

To approximate clinical impact, we derived a pragmatic number needed to evaluate (NNE) using the 14-day re-presentation rate among false-positive admissions (GPT-4o recommended admission, clinician discharged). As shown in Supplementary Table 4, the estimated NNEs were 4.2 (Model 1), 3.2 (Model 2), and 3.4 (Model 3), suggesting that roughly three to four additional GPT-prompted admissions would capture one patient who re-presented within 14 days. These findings are exploratory and should be interpreted cautiously, as re-presentation does not necessarily equate to unsafe discharge.

## Discussion

LLMs such as GPT-4o are increasingly explored as decision support tools in emergency care, particularly for triage and disposition [6, 7]. In this study, we assessed GPT-4o’s diagnostic accuracy in predicting disposition outcomes for patients with isolated pulmonary conditions. Sensitivity improved as input complexity increased, particularly in identifying patients requiring hospital or ICU admission. This was accompanied by reduced specificity and limited accuracy in discharge prediction, consistent with the conservative triage bias described in prior LLM evaluations [5, 8].

Although Models 2 and 3 incorporated progressively more complex inputs, their performance did not differ significantly from the simpler Model 1. This may reflect overlapping informational content, limited sample size, and the ceiling effect in a high-acuity cohort, underscoring that the simpler model may be equally practical.

GPT-4o leverages its training on clinical and non-clinical texts to emulate diagnostic reasoning and prioritize probable outcomes. In our study, this translated into high sensitivity for hospital admission (up to 91.9%), especially when laboratory and imaging data were incorporated (Model 3). However, the model’s specificity was low (20.8%), resulting in a conservative bias toward admission, with GPT-4o recommending hospitalization for approximately 70% of patients who were ultimately discharged. This pattern reflects prior findings that LLMs tend to overestimate illness severity, potentially due to the lack of contextual data such as provider gestalt, psychosocial factors, or real-time observation [4, 5, 9]. While such overtriage may increase hospital bed utilization and associated costs, it may also serve as a safety net by reducing the risk of missed deterioration. These findings underscore a trade-off between patient safety and resource efficiency, which must be carefully weighed in future prospective validations.

In our re-presentation analysis, GPT-4o would have recommended admission for nearly 70% of patients who were initially discharged, though fewer than 30% required actual re-hospitalization. About one-quarter returned to the ED within two weeks, suggesting that the model may have identified risk signals not prioritized during real-time decisions. While causality cannot be established, this pattern supports the idea that LLMs could function as secondary screening tools—highlighting borderline discharge cases for closer follow-up or safety-netting [10, 11]. Prospective validation is needed before such tools can be reliably integrated into clinical workflows.

Several prior studies have evaluated LLMs in emergency medicine, with most focusing on simulated cases, triage categorization, or diagnostic accuracy across generic presentations [2–4, 8]. Our study differs in that it applied GPT-4o to real-world patient data involving formal specialty consultations—specifically pulmonology—reflecting higher diagnostic complexity and real-life disposition challenges. Unlike previous studies that used binary classification (e.g., admit vs. discharge), we evaluated three-level disposition outcomes (discharge, ward, ICU), providing a more granular assessment aligned with ED workflows. Furthermore, we incorporated a stepwise input design and linked model predictions to actual patient outcomes, including early ED re-presentations. These methodological features address critical gaps in the current LLM literature and offer a more pragmatic framework for future clinical integration.

Rather than functioning as a standalone triage engine, GPT-4o may be more useful as a “double-check” tool, flagging cases where disposition decisions warrant additional scrutiny. In this role, the model could highlight patients with borderline parameters or high-risk indicators who might otherwise be discharged prematurely. This adjunctive approach aligns with emerging literature emphasizing augmentation rather than replacement of clinician judgment [7, 12].With further validation, such an approach may offer complementary support in real-world emergency care practice.

Despite promising performance in admission prediction, GPT-4o’s clinical utility remains limited. The model functions as a black-box, providing no transparent reasoning to support trust in high-stakes ED environments [12]. It also cannot account for context-rich factors—such as frailty, social support, or clinician intuition—that are often decisive in disposition [9, 13]. These limitations restrict its standalone deployment and underscore the need for human oversight and explainability in real-world use.

Another critical concern involves the generalizability of GPT-4o’s outputs across different healthcare systems. Most LLMs are trained predominantly on English-language data from Western institutions, which may limit their performance in diverse settings such as Türkiye due to differences in terminology, disease patterns, and clinical practices. These gaps can reduce model reliability and diagnostic alignment, particularly in underrepresented regions [12, 14]. As LLMs become increasingly integrated into decision support tools, region-specific fine-tuning and local validation will be important to ensure accuracy and relevance. Without such adaptation, models that perform well in controlled environments may show reduced reliability in diverse real-world contexts.

In conclusion, GPT-4o demonstrated high sensitivity in predicting hospital and ICU admissions when provided with progressively enriched clinical input, supporting its potential utility in stratifying disposition decisions among patients with complex pulmonary conditions in the ED. However, the model’s limited specificity—particularly in discharge prediction—highlights its tendency toward overtriage, which may reduce efficiency if deployed without oversight. By implementing a structured, multi-layered input strategy and linking predictions to real-world outcomes, this study offers a pragmatic framework for evaluating LLM-based tools in emergency decision-making. Future work should focus on prospective validation, region-specific adaptation, and careful integration into clinical workflows to confirm that such systems can be applied safely and meaningfully in frontline care.

### Limitations

This study has several limitations that should be considered when interpreting the findings.

First, this study was conducted at a single tertiary care center in Türkiye, which may limit generalizability to other settings. The cohort represented patients with greater clinical complexity—those requiring both chest CT and pulmonology consultation—and only 21.7% were discharged from the ED. Therefore, our findings are primarily applicable to high-acuity patients with complex pulmonary conditions.

Second, GPT-4o’s predictions were compared to final dispositions but not to real-time decisions by emergency physicians, limiting assessment of concordance with clinician judgment and potentially underestimating discrepancies during dynamic decision-making.

Third, the prompt-based interaction method introduces potential variability in model responses. Although all prompts followed a standardized structure and were submitted under controlled conditions, even subtle changes in input wording can affect LLM behavior. This limitation reflects a broader challenge in the reproducibility of prompt-based AI evaluations. As detailed in Supplementary Table 1, we applied a single standardized prompt across all models without testing alternative structures. This approach ensured consistency and reflects a proof-of-concept baseline; however, future research may explore whether prompt optimization strategies could further improve performance.

Fourth, this study was designed as a proof-of-concept evaluation. The absence of respiratory rate and blood pressure data reflects dataset limitations and restricts direct translatability. These findings should therefore be viewed as exploratory evidence of potential, pending further validation in prospective studies with complete datasets.

Fifth, structured input tiers enabled performance comparison, but statistical testing showed no significant differences in accuracy or F1-score, likely due to sample size or class imbalance. ICU admissions were under 10% (*n* = 20), limiting subgroup or multivariable analyses.

Sixth, our primary objective was to assess how closely GPT-4o could replicate the disposition decisions made by an experienced pulmonologist in actual clinical practice. These decisions were used as reference outcomes, not as gold standards of clinical correctness. Although GPT-4o tended to recommend admission in some patients who re-presented within 14 days, the retrospective design prevents us from determining whether those discharges were truly unsafe. Disposition at re-presentation (admission vs. discharge) was not consistently available in this retrospective dataset; therefore, re-presentation findings are exploratory and should be interpreted with caution.

Finally, as with all retrospective analyses, the study is subject to limitations in data accuracy and completeness. Although rigorous data cleaning and validation procedures were employed, potential documentation biases or unmeasured confounders may have influenced model inputs or outcome assignment.

## Conclusion

In this diagnostic evaluation of complex pulmonary cases requiring specialist consultation and CT imaging, GPT-4o showed high sensitivity but low specificity in predicting disposition. It performed particularly well in identifying hospital and ICU admissions when provided with enriched clinical inputs such as laboratory results and imaging summaries. These findings suggest that GPT-4o may assist in stratifying disposition decisions among patients with complex pulmonary presentations, supporting its potential use as a decision support tool in emergency triage and disposition.

However, the model’s low specificity—particularly for discharge decisions—indicates a conservative tendency that may contribute to overtriage and increased strain on healthcare resources if not carefully managed. By applying a structured, tiered input approach and linking predictions to real-world outcomes, including ED re-presentations, this study provides a pragmatic framework for evaluating LLM performance in specialty-referred, diagnostically complex emergency cases.

Future work should focus on prospective validation across diverse settings, integration with structured electronic health records, and refinement of prompts or model configurations to improve discharge safety and clinical interpretability. Rather than serving as a standalone triage system, GPT-4o may be more appropriately utilized as a supportive tool to flag potentially high-risk discharges, complementing clinician expertise within the context of high-complexity ED care.

## Supplementary Information

Below is the link to the electronic supplementary material.

Supplementary Material 1

Supplementary Material 2

Supplementary Material 3

Supplementary Material 4

## Data Availability

No datasets were generated or analysed during the current study.

## Abbreviations

AI: Artificial Intelligence
CRP: C-Reactive Protein
CT: Computed Tomography
ED: Emergency Department
EHR: Electronic Health Record
ICU: Intensive Care Unit
LLM: Large Language Model
NPV: Negative Predictive Value
PE: Pulmonary Embolism
PPV: Positive Predictive Value
SpO₂: Peripheral Oxygen Saturation
WBC: White Blood Cell Count
