# Supplementary material for: Evaluating GPT-4o for emergency disposition of complex respiratory cases with pulmonology consultation: a diagnostic accuracy study
Source: Scand J Trauma Resusc Emerg Med. 2025 Oct 2;33:159. doi: 10.1186/s13049-025-01475-3 (PMC12492850; doi:10.1186/s13049-025-01475-3)
Supplement: Supplementary file 1 — Supplementary Material 1 [file 13049_2025_1475_MOESM1_ESM.docx]

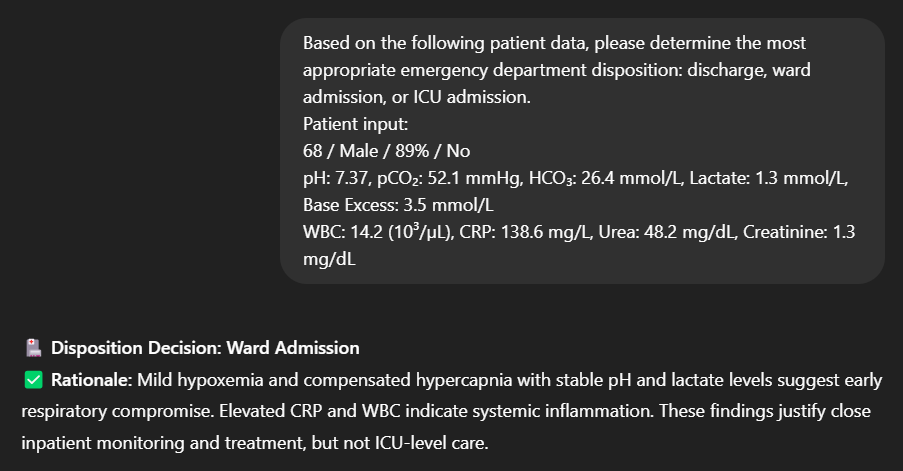


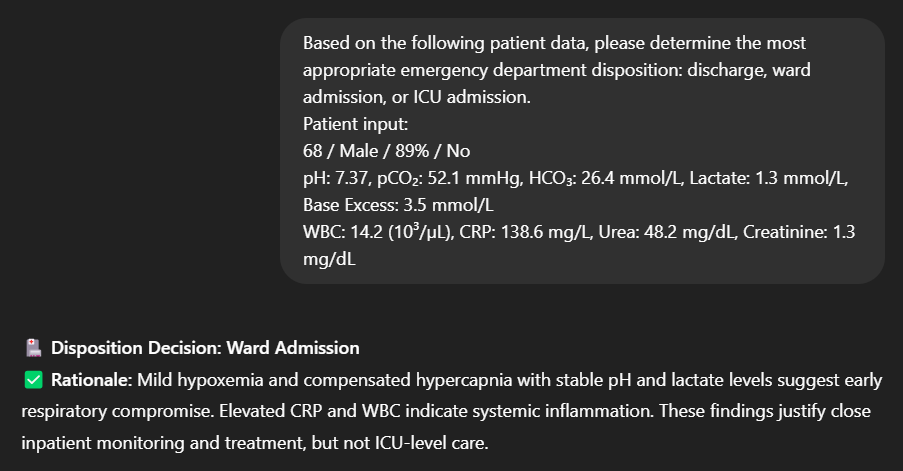


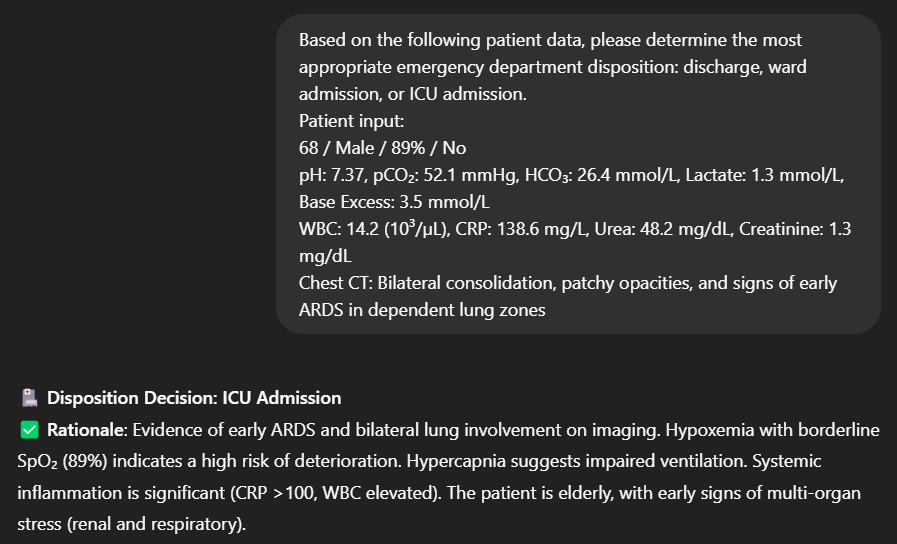


**Supplementary table 1**. Example prompts submitted to GPT-4o for each model.

Patient data are presented in the standardized format:

Age / Sex / SpO₂ / Long-term oxygen therapy (Yes/No), followed by model-specific clinical variables.

(A) Input parameters: Age, Sex, SpO₂, Home O₂ use, pH, pCO₂, HCO₃, Lactate, Base Excess

(B) Input parameters: Model 1 + WBC, CRP, Urea

(C) Input parameters: Model 2 + Chest CT findings
