# Supplementary material for: Evaluating GPT-4o for emergency disposition of complex respiratory cases with pulmonology consultation: a diagnostic accuracy study
Source: Scand J Trauma Resusc Emerg Med. 2025 Oct 2;33:159. doi: 10.1186/s13049-025-01475-3 (PMC12492850; doi:10.1186/s13049-025-01475-3)
Supplement: Supplementary file 2 — Supplementary Material 2 [file 13049_2025_1475_MOESM2_ESM.docx]

| Comparison | Method | Metric Compared | Observed Difference | p-value / 95% CI | Significant? |
| --- | --- | --- | --- | --- | --- |
| Model 1 vs Model 2 | McNemar | Accuracy | – | p = 0.230 | No |
| Model 2 vs Model 3 | McNemar | Accuracy | – | p = 0.815 | No |
| Model 1 vs Model 3 | McNemar | Accuracy | – | p = 0.222 | No |
| Model 1 vs Model 2 | Bootstrap | Accuracy | +3.17% | −1.36% to +7.69% | No |
| Model 2 vs Model 3 | Bootstrap | Accuracy | +0.90% | −2.71% to +4.52% | No |
| Model 1 vs Model 3 | Bootstrap | Accuracy | +4.07% | −1.81% to +9.95% | No |
| Model 1 vs Model 2 | Permutation | F1 Score | +0.033 | p = 0.978 | No |
| Model 2 vs Model 3 | Permutation | F1 Score | +0.013 | p = 0.995 | No |
| Model 1 vs Model 3 | Permutation | F1 Score | +0.046 | p = 0.999 | No |

**Supplementary Table 2**. Comparative Statistical Testing of Model Performance

Comparative statistical analyses between GPT-4o input models (Model 1 vs. Model 2 vs. Model 3) for hospital disposition prediction performance. Pairwise differences in accuracy and F1 score were evaluated using McNemar’s exact test, bootstrap resampling (n = 10,000), and permutation testing (n = 10,000). Confidence intervals (CIs) are bias-corrected and accelerated (BCa). No statistically significant differences were observed across models (all p > 0.05).
