# Supplementary material for: Evaluating GPT-4o for emergency disposition of complex respiratory cases with pulmonology consultation: a diagnostic accuracy study
Source: Scand J Trauma Resusc Emerg Med. 2025 Oct 2;33:159. doi: 10.1186/s13049-025-01475-3 (PMC12492850; doi:10.1186/s13049-025-01475-3)
Supplement: Supplementary file 3 — Supplementary Material 3 [file 13049_2025_1475_MOESM3_ESM.docx]

|  | Subgroup(n) | Model | Accuracy (%) | Sensitivity (%) | Specificity (%) |
| --- | --- | --- | --- | --- | --- |
| Age | <65(72) | Model 1 | 68.1 | 66.1 | 75.0 |
|  |  | Model 2 | 76.4 | 82.1 | 56.2 |
|  |  | Model 3 | 75.0 | 87.5 | 31.2 |
|  | ≥65(149) | Model 1 | 75.2 | 82.2 | 48.4 |
|  |  | Model 2 | 75.8 | 88.1 | 29.0 |
|  |  | Model 3 | 77.9 | 94.1 | 16.1 |
| SpO_2_ | <80% (99) | Model 1 | 92.9 | 100.0 | 0.0 |
|  |  | Model 2 | 92.9 | 100.0 | 0.0 |
|  |  | Model 3 | 92.9 | 100.0 | 0.0 |
|  | ≥80% (122) | Model 1 | 56.6 | 51.2 | 67.5 |
|  |  | Model 2 | 62.3 | 70.7 | 45.0 |
|  |  | Model 3 | 63.9 | 82.9 | 25.0 |

**Supplementary Table 3.** Subgroup accuracy, sensitivity, and specificity by age and SpO₂.

Outcome = hospital admission (ward or ICU).

Cut-offs were chosen for clinical and dataset reasons: ≥65 years is a conventional threshold in respiratory/critical care research, and SpO₂ <80% matched the cohort median. Accuracy was stable across age groups, while hypoxemia (<80%) was associated with high sensitivity but minimal specificity.
