# Supplementary material for: Evaluating GPT-4o for emergency disposition of complex respiratory cases with pulmonology consultation: a diagnostic accuracy study
Source: Scand J Trauma Resusc Emerg Med. 2025 Oct 2;33:159. doi: 10.1186/s13049-025-01475-3 (PMC12492850; doi:10.1186/s13049-025-01475-3)
Supplement: Supplementary file 4 — Supplementary Material 4 [file 13049_2025_1475_MOESM4_ESM.docx]

| Model | False-positive cases (n) | Re-presentations n (%) | Pneumonia  n (%) | COPD  n (%) | COPD+Pneumonia n (%) | ILD  n (%) | NNE  (≈) |
| --- | --- | --- | --- | --- | --- | --- | --- |
| Model 1 | 21 | 5 (23.8%) | 2 (40.0%) | 1 (20.0%) | 1 (20.0%) | 1 (20.0%) | 4.2 |
| Model 2 | 30 | 9 (30.0%) | 6 (66.7%) | 1 (11.1%) | 1 (11.1%) | 1 (11.1%) | 3.2 |
| Model 3 | 38 | 11 (28.9%) | 8 (72.7%) | 1 (9.1%) | 1 (9.1%) | 1 (9.1%) | 3.4 |

**Supplementary Table 4.** Diagnosis distribution among false-positive admissions who re-presented within 14 days.

NNE (number needed to evaluate) was calculated as the reciprocal of the 14-day re-presentation rate among false-positive admissions (GPT-4o recommended admission but the patient was discharged), i.e., NNE = 1 / re-presentation rate.

False positives were defined as cases where GPT-4o predicted admission but the patient was discharged by the pulmonologist. Re-presentations were most frequently associated with pneumonia, followed by COPD, COPD+pneumonia, and interstitial lung disease. These findings highlight that many “false-positive” admissions captured patients who later required repeat ED evaluation.
